# A proof-of-concept study for the design of a VLP-based combinatorial HPV and placental malaria vaccine

**DOI:** 10.1038/s41598-019-41522-5

**Published:** 2019-03-27

**Authors:** Christoph M. Janitzek, Julianne Peabody, Susan Thrane, Philip H. R. Carlsen, Thor G. Theander, Ali Salanti, Bryce Chackerian, Morten A. Nielsen, Adam F. Sander

**Affiliations:** 1 Centre for Medical Parasitology at the Department of Immunology and Microbiology, University of Copenhagen, Copenhagen, Denmark; 2 Department of Infectious Diseases, Copenhagen University Hospital, Copenhagen, Denmark; 3 Department of Molecular Genetics & Microbiology, University of New Mexico School of Medicine, Albuquerque, USA

## Abstract

In Africa, cervical cancer and placental malaria (PM) are a major public health concern. There is currently no available PM vaccine and the marketed Human Papillomavirus (HPV) vaccines are prohibitively expensive. The idea of a combinatorial HPV and PM vaccine is attractive because the target population for vaccination against both diseases, adolescent girls, would be overlapping in Sub-Saharan Africa. Here we demonstrate proof-of-concept for a combinatorial vaccine utilizing the AP205 capsid-based virus-like particle (VLP) designed to simultaneously display two clinically relevant antigens (the HPV RG1 epitope and the VAR2CSA PM antigen). Three distinct combinatorial VLPs were produced displaying one, two or five concatenated RG1 epitopes without obstructing the VLP’s capacity to form. Co-display of VAR2CSA was achieved through a split-protein Tag/Catcher interaction without hampering the vaccine stability. Vaccination with the combinatorial vaccine(s) was able to reduce HPV infection *in vivo* and induce anti-VAR2CSA IgG antibodies, which inhibited binding between native VAR2CSA expressed on infected red blood cells and chondroitin sulfate A in an *in vitro* binding-inhibition assay. These results show that the Tag/Catcher AP205 VLP system can be exploited to make a combinatorial vaccine capable of eliciting antibodies with dual specificity.

## Introduction

There is a striking overlap in the global prevalence of cervical cancer and malaria, particularly in Sub-Saharan Africa. Cervical cancer is caused by a sexually acquired infection with certain types of Human Papillomavirus (HPV)^1^, and causes more than 60,000 deaths annually in sub-Saharan Africa alone^2^. This region also accounts for 86% of global malaria cases (219 million), including 435,000 malaria related deaths per year^3^. Women are particularly vulnerable to malaria during pregnancy when *P. falciparum* infections cause placental malaria (PM) (reviewed by^4^ and^5^). Consequently, both cervical cancer and PM constitute major public health challenges in Sub-Saharan Africa, where people often have limited access to health care (e.g. for regular HPV screening or malaria testing), or the affected individuals may be incapable of paying for expensive vaccines or treatment^2,6^.

HPV vaccination programmes have been widely implemented in the developed world, which has caused a substantial decrease in the incidence of HPV infections^2,7^. The HPV vaccines based on the HPV L1 major capsid protein, which form Virus-Like Particles (VLPs), are extremely potent, possibly offering life-long protection after a single vaccine dose^8,9^. The high immunogenicity of the HPV vaccines is attributed to structural features of the L1 VLPs, which lead to the efficient generation of long-lived antigen-specific antibody-producing cells^10–12^. HPV L1 VLP vaccines strongly elicit neutralizing antibody responses against the included HPV genotypes represented in the vaccines, but offer little in the way of cross-protection against other HPV types (reviewed by^13^). The clinical data generated by the HPV L1 VLP vaccines has consequently stimulated a great interest in developing next-generation VLP-based vaccines with a similar capacity of inducing long-term antibody responses, but against a broader panel of HPV types.

Current efforts to develop a broadly protective prophylactic HPV vaccine focus on targeting the HPV minor capsid protein, L2, which contains multiple highly-conserved and broadly neutralizing epitopes (as reviewed by^14^). In particular, the N-terminus of the HPV L2 protein contains a major cross-neutralizing epitope, RG1^15^. Several studies have shown anti-RG1 antibodies confer complete type-specific protection and moderate cross-protection against heterologous HPV types^16,17^. Recently, it was shown that displaying multiple conjoined L2 RG1 epitopes, deriving from different HPV types on the surface of a VLP backbone, broadened the protective immunity against diverse HPV types^18^. The RG1 epitope, specifically concatenated constructs comprised of the RG1 epitope from several HPV types, represents a good target antigen for the development of a broadly protective HPV vaccine. However, the RG1 epitope is only transiently exposed to the immune system during the attachment of HPV to the basement membrane^19^ and therefore, an RG1-based vaccine will likely need to elicit long-lived, high levels of circulating antibodies to be effective.

To date, there is no commercially available vaccine against PM, although two different vaccines, both including the minimal binding region of the *P. falciparum* adhesion protein, VAR2CSA, are being tested in clinical phase I trials (NCT02647489 and NCT02658253). VAR2CSA is responsible for the sequestration of *P. falciparum* parasites^20^ in the placenta of pregnant women by mediating binding to the placental chondroitin sulfate A (CSA)^21^. Accordingly, an effective VAR2CSA-based PM vaccine may need to elicit antibodies that inhibit the binding between placental CSA and VAR2CSA expressed on the surface of infected erythrocytes (iE)^22–24^.

Vaccines against PM and HPV have overlapping target populations and need to be inexpensive in order to be broadly employed in the sub-Saharan region^25^. Both vaccines should ideally be administered to pre-pubescent girls and elicit durable (decades-long) immune responses. Combining the two disease targets on a common, highly immunogenic VLP platform could be a strategy to induce long-term protection against both diseases, and will be beneficial in terms of production costs and deployment.

We have used the well-characterized AP205 capsid-based VLP platform^26–30^ to generate VLP based vaccines (coined 5x, 2x, and 1xL2-VLPs) that display both VAR2CSA and HPV L2 RG1 epitopes at high densities. Using mouse models, this study examined whether the VLP-based combinatorial vaccine design would allow for induction of high titered, dual-specificity antibody responses against the co-displayed antigens.

## Results

### Engineering 5x, 2x and 1xL2-VLPs

**Figure 1:**
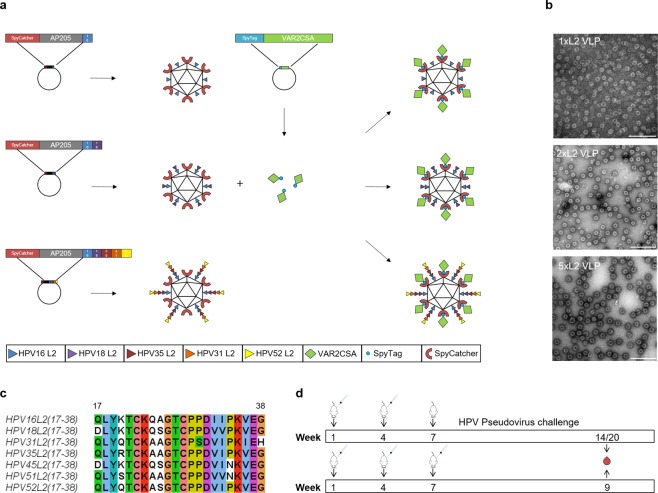
(**a**) Schematic representation of the method used to create the combinatorial HPV and PM VLP vaccines. Three combinatorial HPV and PM VLP vaccines were created. Specifically, the AP205 capsid protein was genetically fused to SpyCatcher at the N-terminus whereas the C-terminus was genetically fused to either one (HPV16), two (HPV16 and 18) or five (HPV16, 18, 35, 31, 52) concatenated peptides derived from the highly conserved, cross-reactive epitope of the HPV L2 minor capsid protein (amino acid 17–38). Recombinant expression in E. coli resulted in formation of three distinct VLPs each displaying 180 L2 polypeptides and SpyCatcher proteins. Subsequent mixing of the PM antigen, VAR2CSA (genetically fused to SpyTag at the N-terminus) with VLPs resulted in covalent attachment of VAR2CSA to the surface of the VLPs. (**b**) Transmission electron microscopy images of the 1xL2-VLP, 2xL2-VLP and 5xL2-VLP. To verify the overall quality of the purified VLPs, the VLP samples were placed on carbon, adsorbed to a grid and negatively stained with 2% uranyl acetate. The three VLPs are non-aggregated and uniform in size (approximately 30–35 nm). Scale bar 200 nm. (**c**) Amino acid sequence alignment of the minor capsid protein L2 (aa 17–38) from HPV16, 18, 31, 35 and 52. The alignment was created using Jalview^36^ under a creative commons license (https://creativecommons.org/licenses/by-sa/3.0/legalcode). (**d**) Mouse immunization schedule. Top panel: Balb/C mice were immunized intramuscularly in a prime-boost regimen (week 1 and week 4) with an antigen dose of 5 µg (based on L2-VLP amount) consisting of either 5xL2-VLP (n = 25), 2xL2-VLP (n = 25), 5xL2-VLP-Var2 (n = 6) or 2xL2-VLP-Var2 (n = 6) VLPs. Three weeks following the last immunization, randomized groups of mice (n = 5) were infected vaginally with luciferase-expressing HPV pseudovirions. Blood samples collected at week 14 (for 5xL2-VLP-Var2 and 2xL2-VLP-Var2) or 20 (for 5 × -L2-VLP and 2xL2-VLP) were analyzed for anti-HPV L2 and anti-VAR2CSA IgG antibodies, both with regards to ELISA binding titers as well as *in vitro* parasite binding-inhibition. Lower panel: Balb/C mice were immunized intramuscularly in a prime-boost-boost regimen (weeks 1, 4 and 7) with a 3.14 µg antigen dose (based on L2-VLP amount) of 1xL2-VLP (n = 7). Blood samples were collected at week 9 to perform ELISA and *in vitro* PsV challenge.

To produce VLPs which simultaneously display the L2-HPV and the VAR2CSA-*P. falciparum* protein, the SpyCatcher sequence and L2-HPV coding sequences were genetically fused to the 5′ and 3′ ends of the gene coding for AP205 capsid protein, respectively (Fig. 1a). The engineered genes were expressed in *E. coli* and the recombinant proteins spontaneously formed VLPs (Fig. 1b). Three different VLPs were produced. The SpyCatcher was fused at the N-terminus of the AP205 capsid protein, but the C-terminus was extended with either one (HPV16), two (HPV16 and 18) or five (HPV16, 18, 35, 31, 52) concatenated peptides derived from the highly conserved, cross-reactive RG1 epitope of the HPV L2 minor capsid protein (amino acid (aa) 17–38) (Fig. 1, panels a–c). The three SpyCatcher/L2-VLPs (termed 1xL2, 2x or 5xL2-VLP) were non-aggregated, had similar diameters (30–35 nm), and had low polydispersity (5xL2-VLP 47.4 nm with 10.1%Pd1, 2xL2-VLP, 45.1 nm with 21.9%Pd and 1xL2-VLP 40.4 nm with Pd% 6.9). The final yield of each VLPs was in the range of 25–100 mg/L culture and the VLPs were stable in 4 C for 14 days and in −80 °C for at least 12 months (Supplementary Fig. S1).

In parallel, the 5′ end of VAR2CSA was genetically fused to SpyTag and expressed in *E. coli*. Mixing of recombinant SpyTag-VAR2CSA protein and the VLPs (*i.e*. 5xL2 or 2xL2), allowed for covalent attachment of VAR2CSA to the VLPs resulting in the formation of VLPs displaying both the HPV L2 and VAR2CSA antigens at high density (Fig. 1, and Supplementary Fig. S2). The combinatorial VLPs are stable and not aggregation prone (Supplementary Fig. S1).

### Immunogenicity of the combinatorial HPV and PM VLP vaccines

**Figure 2:**
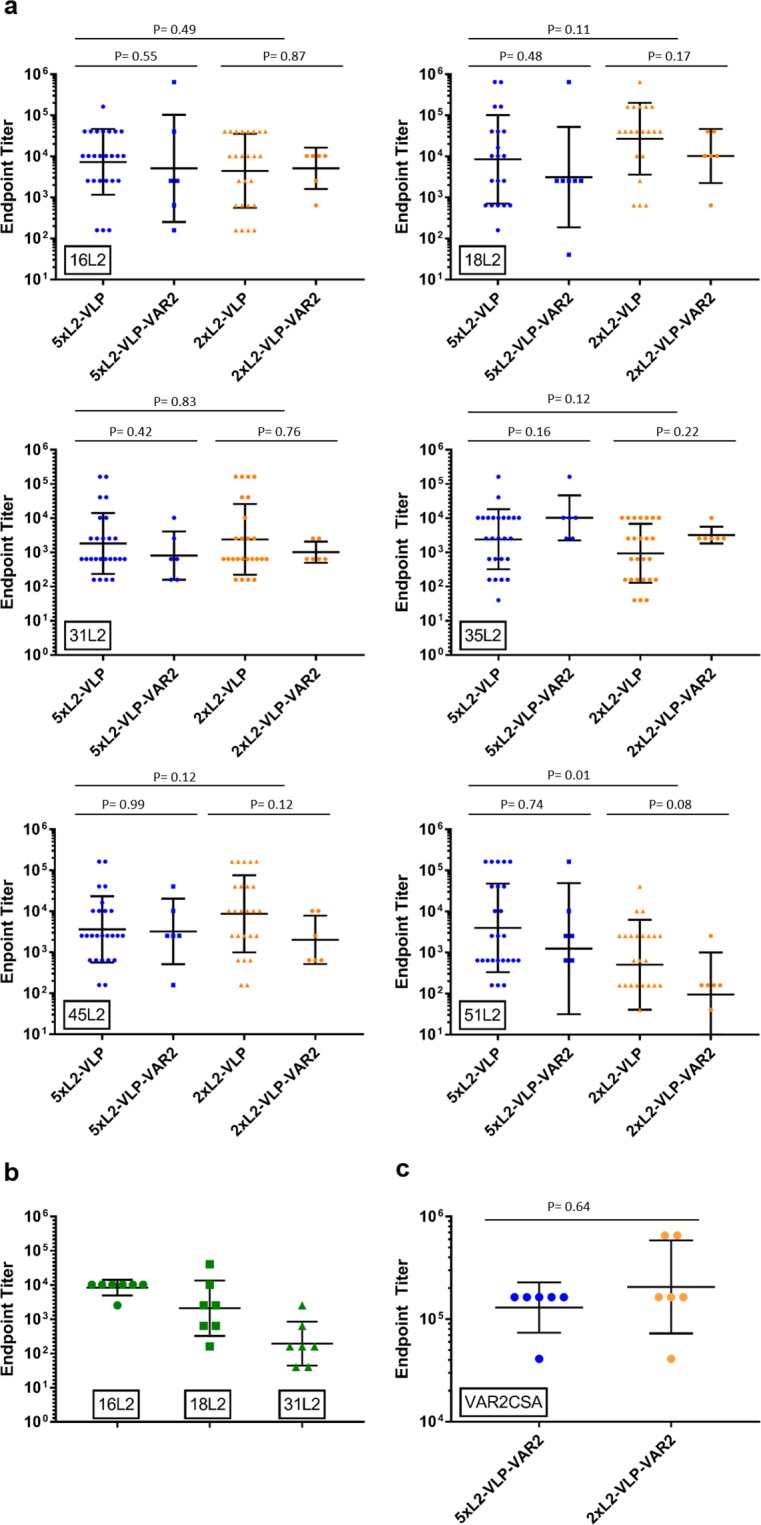
HPV and PM specific endpoint ELISA IgG binding titers. Endpoint antibody binding titers from individual mouse sera were calculated using a cut-off of O.D. = 0.1. Graphs show geometric mean and SD. Mann-Whitney Rank sum test was used for statistical analysis using significance cut-off value of 0.05. (**a**) Vaccine induced anti-HPV L2 binding titers measured in sera from mice immunized with 5xL2- and 2xL2 VLPs (with and without co-display of VAR2CSA). Peptide sequences derived from the HPV L2 RG1 epitope (aa 17–31) of a range of HPV types (*i.e*. 16, 18, 32, 35, 45, and 51) were genetically fused to a carrier protein (accession number WP_057363222, aa 44–338) and used as capture proteins in ELISA. (**b**) Vaccine induced anti-HPV16, 18 and 31 L2 IgG titers, measured by HPV specific ELISA. Mice immunized with 1xL2-VLPs. For measuring HPV type specific antibody responses ELISA plates were coated with synthetic peptides derived from the HPV L2 RG1 epitope (aa 17–31) of HPV types 16, 18 or 31. (**c**) Vaccine induced anti-VAR2CSA IgG titers measured in sera from mice immunized with 5xL2-VLP-VAR2CSA and 2xL2-VLP-VAR2CSA. VAR2CSA was used as capture protein for the ELISA.

The immunogenicity of the combinatorial VLP vaccines was assessed in mice, as described in Fig. 1d, and antibody responses were assessed by ELISA. All three vaccines elicited strong antibody responses against HPV L2. The 1xL2-VLP vaccine elicited antibodies with reactivity against the L2 peptides representing each of the HPV types we tested (16, 18 and 31) (Fig. 2b). As expected, antibody titers were high against the HPV16 L2 RG1 epitope included in the vaccine. The cross-reactivity against the HPV18 L2 was also high while cross-reactivity towards the HPV31 L2 RG1 epitope was lower. The 2xL2-VLP and 5xL2-VLP vaccines also induced antibodies with broad reactivity against the HPV L2 RG1 epitopes tested (*i.e*. 16, 18, 31, 35, 45 and 51) (Fig. 2a). The levels of anti-L2 IgG antibodies elicited by vaccines with and without co-display of VAR2CSA were comparable, indicating that co-display of VAR2CSA does not significantly reduce the anti-HPV L2 antibody response. There was no significant difference in the antibody levels against HPV16, 18, 31, 35, 45 L2 peptides elicited by the 5xL2 and 2xL2-VLP vaccines despite the fact that the 2xL2-VLP vaccine only contained the HPV16 and 18 L2 sequences. However, the 5xL2-VLP induced antibodies showing significantly higher reactivity to the HPV51 L2 RG1 epitope than the 2xL2-VLP (Fig. 2b). Finally, the combinatorial VLP vaccines (2xL2-VLP-VAR2CSA 5xL2-VLP-VAR2CSA) elicited high levels of anti-VAR2CSA IgG, confirming that the AP205 VLP platform can be utilized to generate immune responses against two distinct pathogenic targets simultaneously (Fig. 2c).

### Neutralization capacity of vaccine-induced anti-HPV L2

**Figure 3:**
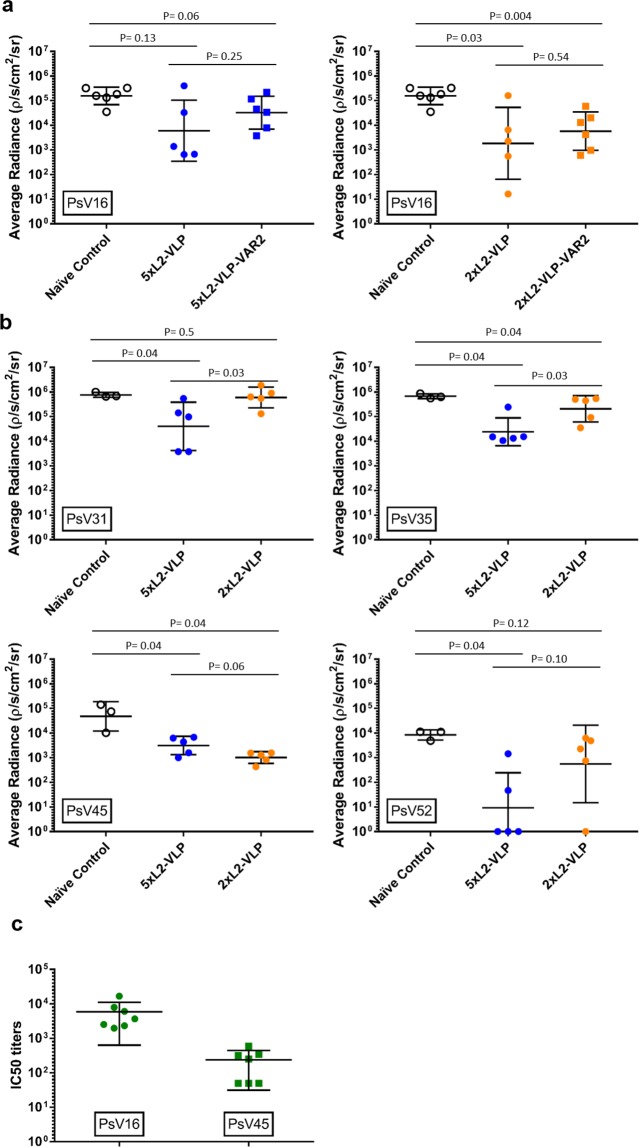
Neutralization capacity of vaccine-induced anti-HPV L2 antibody responses measured *in vivo* PsV genital challenge mouse model and *in vitro* L2-based PsV inhibition assay. Balb/C mice were first immunized and then challenged with HPV PsV, as described in Fig. 1d (top panel). Infection levels in vaccinated mice were measured against non-vaccinated i.e. naïve control mice. Graph shows average radiance for individual mice as well as geometric mean and SD for each group. (**a**) PsV infection in mice immunized with either the 5xL2-VLP (left panel) vaccine or the 2xL2-VLP vaccine (right panel) (with/without co-display of VAR2CSA) after challenge with HPV PsV16. (**b**) PsV infection in 5xL2-VLP or 2xL2-VLP immunized mice challenged with HPV PsV31, 35, 45, or 52. (**c**) Balb/C mice were immunized with 1xL2-VLP, as described in Fig. 1d (lower panel). Neutralization titers (IC50) against HPV PsV 16 and 45 were measured in mouse serum collected 9 weeks after prime vaccination using an *in vitro* L2-based PsV inhibition assay, as previously described^35^.

To test the capacity of vaccine-induced antibodies to neutralize HPV infection *in vivo*, mice immunized with 2xL2-VLP or 5xL2-VLP were challenged with a panel of luciferase expressing HPV pseudovirions (PsV) (*i.e*. PsV16, 31, 35, 45, 52) using a well-established PsV genital challenge mouse model^17,18^ (Fig. 3a,d). In this model the level of infection is indirectly determined by measuring the amount of light (radiance) emitted upon instilling luciferin. First, to test whether co-display of VAR2CSA affected the neutralization capacity of the vaccine-induced anti-HPV L2 antibody response, infection levels in mice immunized with L2-displaying VLPs with or without co-display of VAR2CSA were compared. There was no statistically significant difference in infection levels between mice immunized with L2-VLPs or with L2-VAR2CSA-VLPs (P = 0.25 and 0.54 for 5xL2-VLP and 2xL2-VLP, respectively) (Fig. 3a left and right panel). Further, mice vaccinated with the 2xL2-VLP vaccines showed a significant 84- and 27-fold reduction in infection levels compared to non-vaccinated control mice (P = 0.03 2xL2-VLP; P = 0.004 2xL2-VLP-VAR2CSA) (Fig. 3a, right panel). Although a similar trend could be seen in the 5xL2-VLP vaccinated mice, this difference was not significant (26-fold reduction P = 0.13 5xL2-VLP; 5-fold reduction P = 0.06 5xL2-VLP-VAR2CSA) (Fig. 3a, left panel).

To investigate whether the breadth of protection against HPV infection was increased by adding L2 RG1 epitopes representing additional HPV types to the VLP vaccine design, infection levels in 5xL2-VLP and 2xL2-VLP vaccinated mice, were compared after challenging with PsV31, 35, 45, or 52. This comparison showed that vaccination with 5xL2-VLP vaccines resulted in a statistically significant, 15-to 916- fold reduction in infection levels of all included PsV types (P = 0.04 for PsV 31, 35, 45 and 52). By contrast, the 2xL2-VLP vaccine were generally less effective than the 5xL2-VLP vaccine at preventing infection with HPV PsV. Vaccination with 2xL2-VLP only caused a significant 3- and 47-fold reduction in PsV35 (P = 0.04) and 45 (P = 0.04) infection levels, respectively while no significant reduction was seen for PsV31 (P = 0.5) and 52 (P = 0.12) infection (Fig. 3b).

Sera from mice immunized with the 1xL2-VLP vaccine were tested in an *in vitro* HPV L2-based PsV inhibition assay. The tested sera showed both homologous PsV16 as well as heterologous neutralizing capacity. As expected, the neutralizing titers against the homologous PsV were higher compared to the heterologous PsV45 neutralization titers. (Fig. 3c).

### Functional capacity of anti-VAR2CSA IgG responses

**Figure 4:**
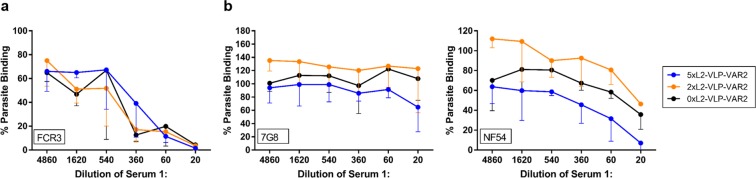
Functional antibody response against VAR2CSA measured *in vitro* by a parasite binding-inhibition assay. A static parasite binding-inhibition assay was used to assess the capacity of vaccine-induced mouse antibodies to prevent binding between native VAR2CSA expressed by iE and the human receptor, chondroitin sulfate A (CSA)^32,33^. In brief, erythrocytes infected with VAR2CSA-expressing tritium-labelled *P. falciparum* were incubated either without serum (positive control) or with mouse sera in 3-fold dilutions starting at 1:20 in decorin-coated wells. Each assay measured mean iE binding and the percentage of binding iE, by dividing the test result with the mean value of iE in wells incubated without serum. Three repetitions of the assay were made for FCR3 and 7G8 and two for NF54. The graphs show the mean percentage of parasite binding across the technical repetitions of the assay. The error bars indicate the standard deviation of this mean. (**a**) Normalized iE binding of the homologous FCR3 strain after pre-incubation with different dilutions of pooled mouse sera of mice immunized with either 5xL2-VLP-VAR2CSA (n = 6, blue), 2xL2-VLP-VAR2CSA (n = 6, orange), or 0xL2-VLP-VAR2CSA (n = 5, black. previously published data^27^ here shown as reference). (**b**) Normalized iE binding of the heterologous 7G8 strain (left) and NF54 strain (right) after pre-incubation with the indicated dilutions of pooled serum samples as in (**a**).

The biological activity of vaccine-induced anti-VAR2CSA IgG was tested using an assay measuring the inhibition of binding between *P. falciparum* infected erythrocytes expressing VAR2CSA and chondroitin sulfate A (CSA) (Fig. 4a). IgG induced by any of the two combinatorial vaccines (5xL2-VLP-Var2 or 2xL2-VLP-Var2) showed binding-inhibitory capacity. The binding inhibition induced by VLPs presenting both L2 and VARCSA, was comparable to the levels induced by VLPs presenting VAR2CSA only (serum from mice already published in^27^ was included in the assay as a reference and presented with the black line 0xL2-VLP-VAR2CSA in these graphs). Inhibition was most pronounced towards parasites of the FCR3 genotype on which the VAR2CSA sequence was based. Some heterologous inhibition was seen towards parasites of the NF54 genotype, but not towards parasites of the 7G8 genotype (Fig. 4b).

## Discussion

The aim of this study was to establish proof-of-concept for a VLP-based combinatorial vaccine eliciting functional antibody responses against both HPV and placental malaria. The unique attribute of the AP205 VLP, to accept genetic fusions at both the N- and C-terminus, was exploited to generate combinatorial VLP vaccines, which were designed to simultaneously display the placental malaria antigen (VAR2CSA) and between one and five concatenated HPV L2 sequences representing the cross-reactive RG1 epitope (aa 17–38). Specifically, L2 RG1 antigen sequences were based on the most prevalent oncogenic HPV types in the sub-Saharan African region.

The first question we sought to answer was whether the large genetic fusions to the AP205 capsid protein would hamper VLP assembly. To our knowledge the largest polypeptide that has previously been fused successfully to the C-terminus of AP205 is 55aa^31^ (NEF55) and co-display of antigenic polypeptides, accomplished by genetic fusion at both N- and C-terminus, has not previously been reported for any VLP. Remarkably, AP205 capsid proteins genetically fused to SpyCatcher (122 aa) at the N-terminus and to HPV L2 RG1 sequences (up to 118aa) at the C-terminus retained their ability to assemble into uniform, non-aggregated VLPs. These results confirm the exceptional capacity of the AP205 VLP to tolerate large genetic fusions at both the N-and C-terminus and underline the broad applicability of this VLP backbone for VLP-based vaccines.

Next, we investigated the ability of the combinatorial VLP based vaccines to generate functional antibody responses against the co-displayed antigen targets. To this end, a theoretical concern of the vaccine design was that one of the co-displayed antigens could dominate the immune response over the other and prevent induction of protective levels of antibodies against the less dominant. In particular, we speculated that covalent attachment of the large PM antigen (VAR2CSA) may sterically hinder access to the RG1 epitopes and/or dominate the immune response due to the high number of B and T-cell epitopes present in the antigen. However, the combinatorial vaccines all elicited high serum levels of anti-RG1 antibodies and co-display of VAR2CSA did not have any measurable effect on the induction of these antibodies. Finally, mice challenged with HPV16 PsV experienced a similar reduction in infection level after immunization with 2xL2-VLP with and without co-display of VAR2CSA. Concerning the PM antigen target, all combinatorial vaccines elicited high levels of anti-VAR2CSA antibodies, which were able to inhibit binding of both homologous and heterologous malaria parasite strains to their human receptor *in vitro*. Collectively, these results support the conceptual design of a VLP-based combinatorial HPV/PM vaccine.

Furthermore, using the PsV *in vivo* challenge model it was demonstrated that immunization with 5xL2-VLPs (comprising RG1 from HPV16, 18, 31, 35, 52) caused significant protection against a broader panel of HPV strains compared to immunization with 2xL2-VLPs (comprising RG1 from HPV16, 18). Whereas both vaccines caused a significant reduction in infection levels of PsV35 and PsV45 only the 5xL2-VLP vaccine induced significant protection against PsV31 and PsV52. Considering the RG1 sequence similarity across the five HPV genotypes included in the 5xL2-VLP vaccine design, HPV35 and HPV45 RG1 sequences show the highest similarity to the corresponding HPV16 and HPV18 sequences, respectively (Fig. 1c). The HPV45 and HPV18 RG1 sequences differ at only a single amino acid position (i.e. position 34) while the HPV16 and HPV35 sequences differ at two positions (i.e. positions 20/24). This could explain why antibody responses induced by the 5xL2-VLP and 2xL2-VLP vaccines are similar both in terms of anti-HPV35/45 antibody binding titers as well as their capacity to neutralize PsV35 and PsV45. In comparison, HPV31 and HPV52 RG1 sequence are less similar to the HPV16/18 RG1 sequences, which may explain why only the 5xL2-VLP vaccine could neutralize PsV of these genotypes. These results were not consistent with the vaccine-induced anti-HPV31 RG1 antibody titers measured by ELISA, which were similar in mice immunized with 5xL2-VLP and 2xL2-VLP vaccines. These data suggest that RG1 sequences may induce a polyclonal mixture of antibodies with different capacities for PsV neutralization.

A final consideration regarding the combinatorial vaccine design relates to the relative order of linked RG1 epitopes displayed on the VLP, which may have an effect on their immunogenicity. Our results showed that mice vaccinated with 5xL2-VLPs, where the HPV16 RG1 epitope was situated closest to the VLP backbone were not protected when challenged by PsV16. This could reflect that HPV16 RG1 was not accessible to B-cell receptors. On that basis, it may be possible to optimize the vaccine-induced anti-RG1 antibody response by changing the order of RG1 epitopes or introducing a longer linker between the VLP backbone and the first RG1 epitope.

In conclusion, our data supports the rational design of a VLP-based combinatorial HPV/PM vaccine with the potential to induce protection against HPV and PM in low-income settings.

## Methods

### Design, expression and purification of 5x, 2x and 1xL2-VLP

The RG1 epitope of HPV16 (QLYKTCKQAGTCPPDIIPKVEG), HPV16 and 18 (QLYKTCKQAGTCPPDIIPKVEG*gs*DLYKTCKQSGTCPPDVVPKVEG) or HPV16, 18, 35, 31 and 52 (QLYKTCKQAGTCPPDIIPKVEG*gs*DLYKTCKQSGTCPPDVVPKVEG*gs*QLYRTCKAAGTCPPDVIPKVEG*gs*QLYQTCKAAGTCPSDVIPKIEH*gs*QLYQTCKASGTCPPDVIPKVEG) was attached to the 3′ of the full-length SpyCatcher-AP205 gene^27^ by PCR. A small Glycine-Serine (GS) linker was inserted between L2 peptides followed by a Glycine-Glycine-Serine-Glycine (GGSG) linker between the SpyCatcher-VLPs and the L2 peptide(s). To create 1xL2-VLPs, the SpyCatcher-AP205 VLP served as template for the initial PCR, where GGSG-16L2 was added by including the nucleotide sequence for the L2 RG1 epitope of HPV16 in the reverse primer overhang.

The 5x and 2xL2-VLPs were created by PCR. The SpyCatcher-AP205 VLP sequence^27^ was amplified with a 5′ NcoI site and a 3′ overlap capable of annealing to the 5′ of a HPV16 RG1 sequence. In the second PCR reaction, a synthetic gene construct (GeneArt, Thermo Scientific) containing the concatenated L2 RG1 epitope of HPV16, 18, 35, 31, 52 was used to create an amplicon with a 5′ overlap, capable of annealing to the 3′ end of the SpyCatcher-AP205 sequence and containing a 3′ NotI cloning site. For 5xL2-VLP, the full sequence was amplified, whereas only the 16 and 18L2 sequences were amplified for creating the 2xL2-VLPs. In a third PCR reaction, the amplicons of the first and second PCR were mixed in order to amplify the 5x and 2xL2-VLP constructs.

The resulting 5xL2, 2xL2 and 1xL2 VLP genes were cloned into a pET-15b vector using NcoI and NotI restriction sites and the SpyCatcher-AP205-L2-pET-15b plasmid was transformed into *E. coli* One Shot® BL21 Star™ (DE3) cells (Thermo Scientific). The non-optimized small-scale upstream expression and downstream purification accomplished using Optiprep™ (Sigma) step gradients, as previously described^27,28^.

### Design, expression and purification of SpyTag-VAR2CSA-HIS antigen

PCR added SpyTag (AHIVMVDAYKPTK) and a flexible GGS-linker 5′, as well as a V5 and hexa-histidine tag (HIS) 3′ to the gene coding for the DBL1-ID2A region (aa 9–1025) of VAR2CSA (FCR3 genotype)^21^ (herein termed VAR2CSA). The SpyTag-VAR2CSA-HIS gene sequence was subcloned into the pET-15b vector as described above, expressed in C3029H SHuffle T7 competent *E.coli* cells (New England Biolabs Inc.) and purified by IMAC and size exclusion chromatography (HiLoad Superdex 200 pg, GE Healthcare).

### Quality assessment of 5x, 2x, and 1x-L2-VLPs

The 5x, 2x and 1x-L2-VLPs were assessed using negative stain transmission electron microscopy (TEM) as well as dynamic light scattering (DLS) as previously described^27,28^. Briefly, for TEM, the 5x, 2x and 1xL2-VLPs were adsorbed to 200-mesh carbon-coated grids. Grids were stained with 2% uranyl acetate (pH7.0) and analyzed with an accelerating voltage of 80 kV, using a CM 100 BioTWIN electron microscope (Phillips, Amsterdam).

For DLS measurements, the distribution of particle sizes was acquired (658 nm, at 25 °C, WYATT Technology, DynaPro NanoStar). The 5x and 2xL2-VLPs were analyzed with 20 runs and the 1xL2-VLP with 10 runs. The estimated diameter of the main particle population and the percent polydispersity (%Pd), was calculated for all three L2-VLPs.

### Conjugation of VAR2CSA to 5x and 2xL2-VLPs

To generate the combinatorial HPV and PM VLP vaccines, purified SpyTag-VAR2CSA-His and 2x or 5xL2-VLPs were mixed in a 3:1 molar ratio (VAR2CSA:VLP) and incubated for 16 h at 4 °C in a phosphate-buffered saline (PBS), pH 8 and adjusted to 0.6 M NaCl.

Excess, unbound antigen was removed by dialysing (1000 kDa molecular weight cut-off, Spectrum Labs) against PBS pH8, 0.6 M NaCl.

### Vaccine formulation

VLP vaccines (5xL2, 2xL2, 1xL2-VLP or the equivalent combinatorial vaccines) were formulated 1 hour prior to immunizations using 2%Alhydrogel (Brenntag) to a final concentration of 2 mg/ml aluminum hydroxide.

### Mouse immunization Studies

8 week old, female BALB/c mice (Taconic, Denmark) were immunized intramuscularly (thigh muscle) with 3.14 µg antigen dose 1xL2 VLPs (based on the total L2-VLP amount in a prime-boost-boost regimen on week 1, 4 and 7 (Fig. 1d lower panel, n = 7). Blood samples were collected at week 9 and the *in vitro* HPV PsV challenge was performed on this serum. For the 5x and 2xL2-VLP vaccines (with and without VAR2CSA), 8 week old, female BALB/c mice (Jackson Laboratories, USA) were immunized with 5 µg (based on the total L2-VLP amount). Mice receiving the 5xL2 (n = 25) or 2xL2-VLP (n = 25) vaccines or the corresponding combinatorial vaccines (n = 6 in each group) were immunized in a prime-boost regimen on week 1 and 4 (Fig. 1d, upper panel). Blood samples were collected at week 5 and at week 14 (for 5xL2-VLP-Var2 and 2xL2-VLP-Var2) and week 20 (for 5 × -L2-VLP and 2xL2-VLP) to analyze HPV/VAR2CSA specific serum IgG titers and to perform the *in vitro* parasite binding-inhibition assay.

### Serum immunoglobulin levels

Enzyme-linked immunosorbent assay (ELISA) was used to detect the level of anti-VAR2CSA and anti-L2 immunoglobulin (IgG) levels. For VAR2CSA specific IgG titers, 96-well microtiter plates (Nunc MaxiSorp) were coated with 0.1 µg/well recombinant VAR2CSA protein (no SpyTag). The L2 peptide (aa 17–31) of the 16, 18, 31, 35, 45 or 51 HPV types was genetically fused to the N-terminus of an unrelated mycobacterium tuberculosis antigen (accession number WP_057363222, aa 44–338). For 5xL2- and 2xL2-VLP studies, the recombinant fusion proteins were used as coat protein for all the ELISAs. For the 1xL2-VLP study, ELISA plates were coated with synthetic L2 peptides (aa 17–31) of the HPV16, 18 or 31, through conjugation of the biotinylated peptides to streptavidin as described elsewhere^18^.

Plates were developed with TMB and signal was measured at O.D.450. An O.D. cutoff 0.1 of was used to determine the endpoint titers of PM and HPV specific serum IgG.

### Parasite binding-inhibition assay

An *in vitro* parasite binding-inhibition assay was employed to determine the capacity of antibodies induced by the 5x, and 2x-VLP-VAR2CSA vaccine to inhibit the binding between native VAR2CSA expressed on iE and CSA (decorin Sigma). For a detailed description of the assay see^32,33^. In short, panning of late-stage *Plasmodium falciparum* (homologous FCR3 or heterologous NF54 and 7G8 genotypes) parasites selected for a CSA-binding phenotype. CSA-binding parasites were labeled with Tritium and added to CSA-coated wells. Diluted, pooled VAR2CSA immune serum (5xL2-Var2 n = 6, 2xL2-Var2 n = 6 and 0xL2-Var2 n = 5) was added in triplicates. Unbound iE were washed off by a pipetting robot (Beckman-Coulter) and remaining iE were collected onto a filter plate (Perkin-Elmer). Liquid scintillation counting (Topcount NXT, Perkin-Elmer) determined the number of iE not inhibited by the VAR2CSA immune sera. Each assay measured mean iE binding and the percentage of binding iE was calculated by dividing the test result with the mean value of iE in wells incubated without serum.

The assay was repeated three times for FCR3 and 7G8 and twice for NF54. The data points represent the mean percentage parasite binding across the technical repetitions of the assay. Error bars represent the standard deviation of this mean. BSA coated wells and naïve sera were used as a negative control.

### Pseudovirion Production

Type 16, 31, 35, 45 and 52 HPV PsV containing a reporter plasmid encoding for both GFP and luciferase were generated using HEK293TT cells as described elsewhere^34^.

The infectious unit (IU) count was determined by infecting HEK293TT cells for 48 h and measuring the percentage of infected (GFP-positive) cells by flow cytometry.

### ***In vitro*** L2 based HPV pseudovirion neutralization assay

The capacity of anti-L2 antibodies to inhibit infection by the homologous HPV16 PsV as well as heterologous HPV45 PsV was determined for sera obtained from mice with 1xL2-VLP vaccine using a previously described L2-based HPV PsV neutralization assay^35^.

### ***In vivo*** HPV Pseudovirion Neutralization Assay

Vaccine induced protection against HPV infection was evaluated in an *in vivo* PsV genital challenge model, as described previously^17,18^. Briefly, Balb/C mice previously immunized with 5xL2-, 2xL2-VLP, or the corresponding combinatorial vaccines, were randomized into challenge groups (n = 5 for 5xL2-VLP and 2xL2-VLP or n = 6 for 5xL2-VLP-Var2, 2xL2-VLP-Var2) starting approximately 3 weeks after the final boost. Five days prior to challenge, mice received 3 mg progesterone. Mice were briefly anesthetized using isoflurane and vaginally challenged with 6.3*10^6^ infectious units (IU) PsV16, 3.15*10^7^ IU PsV31, 8.84*10^6^ IU PsV 35, 1.19*10^7^ IU PsV45 or 6.2*10^5^ IU PsV52. PsV infection was assessed under inhalation anesthesia (isoflurane) 48 hours after challenge by instilling 0.4 mg luciferin vaginally and measuring radiance (five minute exposure) using a Caliper IVIS Lumina II (Caliper Life Sciences). Background radiance was subtracted from this radiance value in order to determine average radiance (p/s/cm2/sr). Immunized mice were compared to groups of unvaccinated mice (n = 6 for PsV16 challenge and n = 3 for PsV31, 35, 45, and 52).

### Ethical Statement

All animal studies were performed in accordance with Federation of European Laboratory Animal Science Associations (FELASA) and American Association for Laboratory Animal Science (AALAS) guidelines and regulations and were approved by the Danish Animal Experiments Inspectorate (Approval number: 2013-15-2934-00902/BES) and the University of New Mexico Institutional Animal Care and Use Committee (IACUC), Animal Welfare Assurance # D16–00228 (A3350-01) USDA Registration # 85-R-0014, Protocol No. 16-200498-HSC.

### Statistical Analysis

GraphPadPrism7.0 was used for calculating geometric mean titers, geometric standard deviation, preparing all graphs and statistical calculations. The non-parametric, two tailed, Mann-Whitney Rank Sum Test was applied in order to determine statistical significance (P < 0.05).

## Supplementary information

Supplementary Dataset 1

## Data Availability

The datasets generated and analyzed during the current study are available from the corresponding author on reasonable request.
