# Supplementary material for: A proof-of-concept study for the design of a VLP-based combinatorial HPV and placental malaria vaccine
Source: Sci Rep. 2019 Mar 27;9:5260. doi: 10.1038/s41598-019-41522-5 (PMC6437161; doi:10.1038/s41598-019-41522-5)
Supplement: Supplementary file 1 — Supplementary Dataset 1 [file 41598_2019_41522_MOESM1_ESM.pdf]

**Supplementary Data**

# A proof-of-concept study for the design of a VLP-based combinatorial HPV and placental malaria vaccine.

Christoph M. Janitzek[1+], Julianne Peabody [2+], Susan Thrane[1], Philip H. R. Carlsen[1], Thor G. Theander[1], Ali Salanti[1], Bryce Chackerian[2], Morten A. Nielsen[1*], Adam F. Sander[1*]

[1] Centre for Medical Parasitology at the Department of Immunology and Microbiology, University of Copenhagen, and Department of Infectious Diseases, Copenhagen University Hospital, Denmark.

[2] Department of Molecular Genetics & Microbiology, University of New Mexico School of Medicine, USA

+These authors have contributed equally to this work.

*Corresponding authors

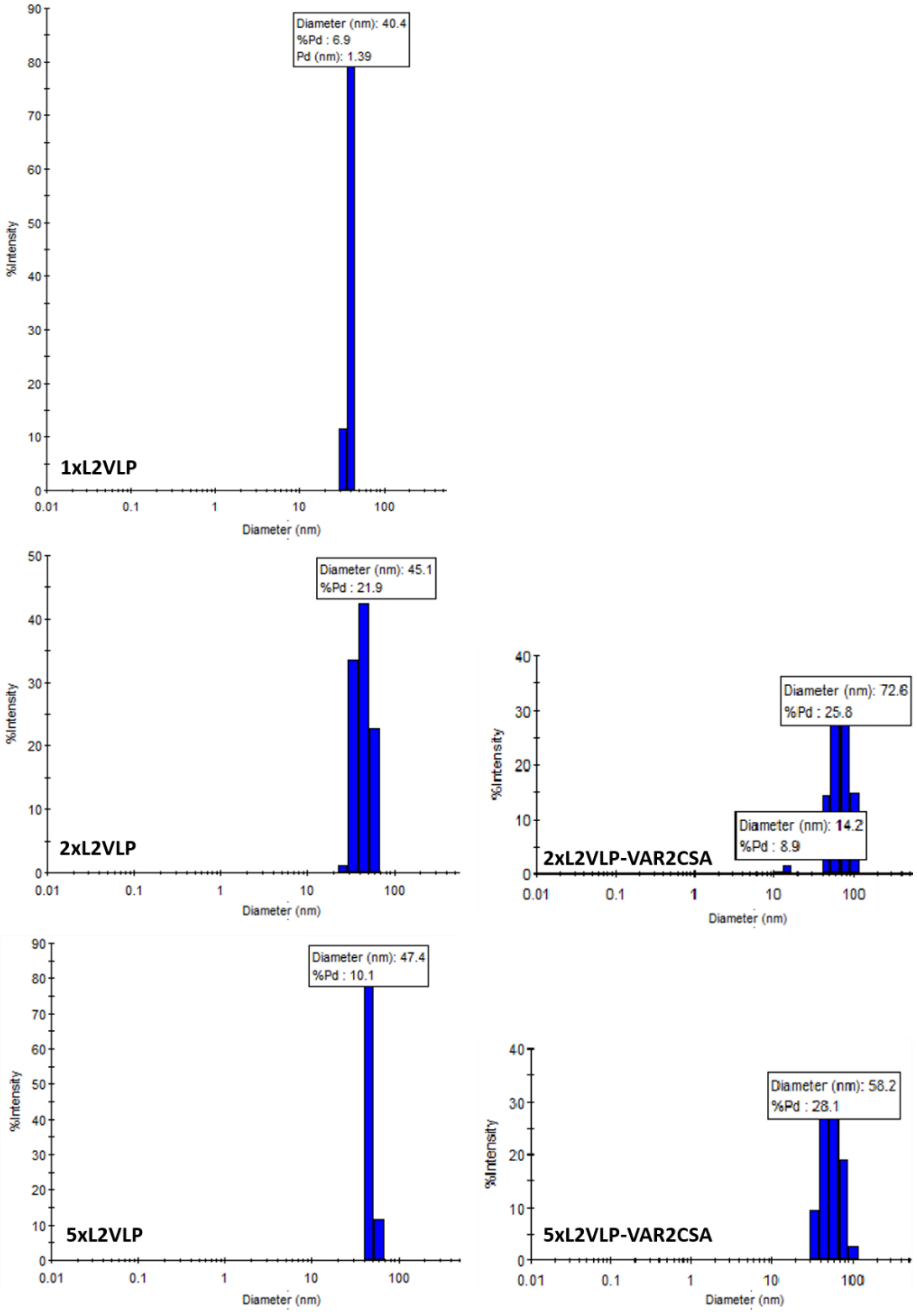


**Supplementary Figure S1** **DLS analysis of L2-VLPs before and after conjugating VAR2CSA.**

The calculated % polydispersity and diameters of the unconjugated L2-VLPs were: 5xL2-VLP 47.4nm with 10.1 %Pd, 2xL2-VLP, 45.1nm with 21.9 %Pd and 1xL2-VLP 40.4nm with Pd% 6.9. The 1xL2-VLP had been stored for 12 months at -80C. The conjugation of VAR2CSA caused the calculated %Pd and diameters to increase (as expected) to 28.1% and 58.2 nm for 5xL2-VLP-VAR2CSA and 25.8% and 72.6nm for 2xL2-VLP-VAR2CSA.

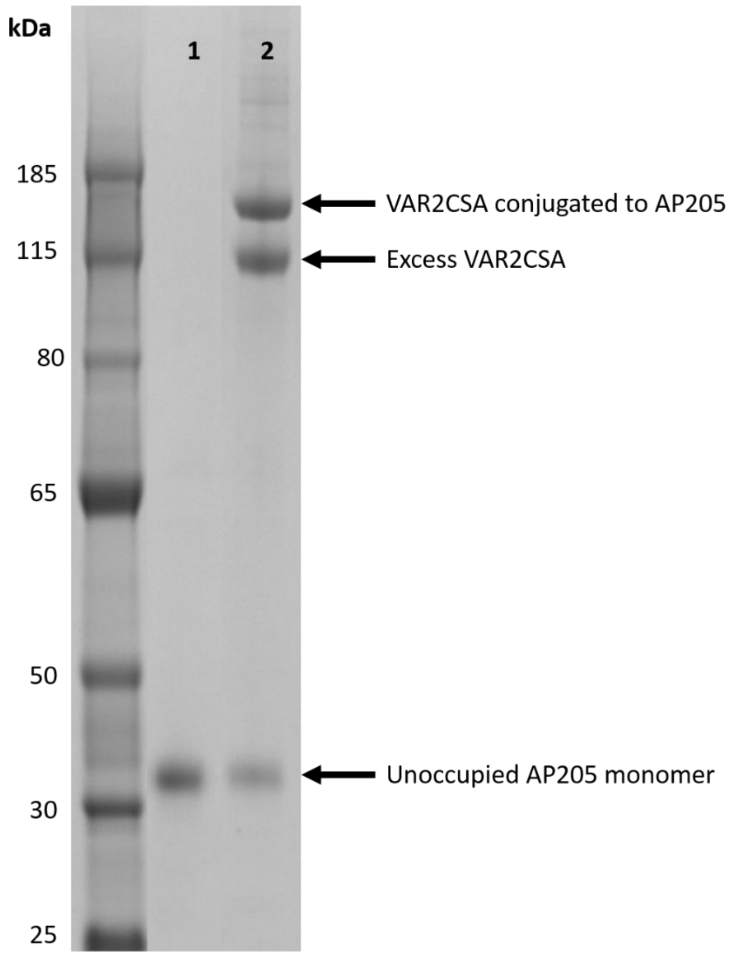


.

**Supplementary Figure S2 Coupling of VAR2CSA to 2xL2-VLP.**

Reduced SDS PAGE gel demonstrating the coupling reaction (using the SpyTag/SpyCatcher interaction) of 2xL2-VLP to VAR2CSA. Lane 1 2xL2-VLP loaded in corresponding amount to the coupling reaction. Lane 2, coupling reaction showing the coupling reaction components, as indicated with arrows. Densitometric analysis of revealed an estimated coupling efficiency (i.e. % VLP subunit coupled to VAR2CSA) of 30%, corresponding to 54 occupied 2xL2-VLP monomers per 2xL2-VLP.
